# Fullerene Rosette: Two-Dimensional Interactive Nanoarchitectonics and Selective Vapor Sensing

**DOI:** 10.3390/ijms23105454

**Published:** 2022-05-13

**Authors:** Guoping Chen, Biswa Nath Bhadra, Linawati Sutrisno, Lok Kumar Shrestha, Katsuhiko Ariga

**Affiliations:** 1 Department of Advanced Materials Science, Graduate School of Frontier Sciences, The University of Tokyo, 5-1-5 Kashiwanoha, Kashiwa 277-8561, Japan; 9290561136@edu.k.u-tokyo.ac.jp; 2 International Center for Materials Nanoarchitectonics (WPI-MANA), National Institute for Materials Science (NIMS), 1-1 Namiki, Tsukuba 305-0044, Japan; sutrisno.linawati@nims.go.jp

**Keywords:** fullerene, formic acid, quartz crystal microbalance, rosette, self-assembly, sensor, two-dimensional material, volatile organic compound

## Abstract

The simplicity of fullerenes as assembled components provides attractive opportunities for basic understanding in self-assembly research. We applied in situ reactive methods to the self-assembly process of C_60_ molecules with melamine/ethylenediamine components in solution, resulting in a novel type of fullerene assemblies, micron-sized two-dimensional, amorphous shape-regular objects, fullerene rosettes. ATR–FTIR spectra, XPS, and TGA results suggest that the melamine/ethylenediamine components strongly interact and/or are covalently linked with fullerenes in the fullerene rosettes. The broad peak for layer spacing in the XRD patterns of the fullerene rosettes corresponds roughly to the interdigitated fullerene bilayer or monolayer of modified fullerene molecules. The fullerene rosettes are made from the accumulation of bilayer/monolayer assemblies of hybridized fullerenes in low crystallinity. Prototype sensor systems were fabricated upon immobilization of the fullerene rosettes onto surfaces of a quartz crystal microbalance (QCM), and selective sensing of formic acid was demonstrated as preliminary results for social-demanded toxic material sensing. The QCM sensor with fullerene rosette is categorized as one of the large-response sensors among reported examples. In selectivity to formic acids against basic guests (formic acid/pyridine >30) or aromatic guests (formic acid/toluene >110), the fullerene rosette-based QCM sensor also showed superior performance.

## 1. Introduction

As commonly seen in functional biological systems such as photosynthetic systems and signal transduction systems, assembled structures often provide much higher properties than each component molecule [1,2,3]. Therefore, molecular self-assembly is regarded as one of the desirable molecular sciences from viewpoints of structural controls and function developments [4,5,6]. Among countless research efforts on the self-assembly of variously designed component molecules, self-assembly research with fullerenes and fullerene derivatives has particular importance [7,8,9]. In addition to the functional potentials of fullerenes due to their unique electronic natures, their simplicity as assembled components provide attractive opportunities for basic understanding in self-assembly research. For example, a representative fullerene molecule, C_60_, is made with a single element, carbon, and has a symmetric spherical shape. Fullerenes are very basic units for self-assembly with features of one-element composition and ideal zero-dimensional shape.

Contrary to the highest simplicity of unit components, the self-assembling process of fullerene molecules creates huge varieties of assembled shapes, as actively reported [10,11,12]. The self-assembly processes of fullerenes and their derivatives can be categorized into two types. First, unmodified fullerenes such as C_60_ and C_70_ molecules are assembled into variously shaped crystals [13,14,15]. In the liquid–liquid interfacial precipitation methods [16], fullerene crystals are precipitated based on solubility differences between two kinds of solvents. Although its methodology is simple, alterations of precipitation conditions, mainly with solvent combinations, provide self-assembled fullerene crystals in a wide range of shapes, including whiskers [17,18], rods [19,20], tubes [21,22], two-dimensional regular-shaped sheets [23,24,25], cubes [26,27], and others [28,29]. More complicated and hierarchical structures such as hole-on-cubes [30], rods-on-cubes [31], macaroni-shaped objects [32], and microhorns [33] are obtained through dynamic processes and post-solvent treatments. All these objects are fundamentally formed through crystallization.

Another type of fullerene assembly is based on the amorphous assembly to give relatively soft objects. Modified fullerenes often behave similar to lipids and amphiphiles to form cell-membrane-like bilayer structures [34,35]. In a pioneering example by Chu, Nakamura, and coworkers, introducing the potassium salt of the pentaphenyl group makes C_60_ fullerene molecules amphiphilic. It forms cell-like spherical vesicular structures in water [36]. Modifying fullerene molecules with alkyl chains results in aromatic–aliphatic amphiphilicity to form variously shaped amorphous assembles, as reported by Nakanishi et al. [37]. These assembled structures have soft and flexible natures. For example, a mixed assembly of modified fullerenes exhibited time-programmed shape-shifting as supramolecular differentiations from egg-type assemblies to tadpole-like objects [38]. Alkylated fullerene molecules with selected alkyl chains show liquid phases at room temperatures [39].

Instead of using pre-modified fullerenes, post-modification of fullerene components in fullerene crystal assemblies can induce drastic morphology changes. Based on the active reactivity of fullerenes with certain amine reagents such as ethylenediamine, fullerene crystals with defined shapes such as rods, hexagons, and cubes are chemically etched into structures with integrated inside pores, simply with addition of ethylene diamine [40]. Recently, this in situ interactive method with ethylenediamine has been coupled with self-assembly processes at the liquid–liquid interface to provide a widely expanded two-dimensional sheet with micropores of amine-reacted C_60_ molecules [41]. Although the latter in situ reactive self-assembly has high potential in the preparation of highly integrated structures and nitrogen-doped materials, this strategy has not been fully explored.

**Figure 1:**
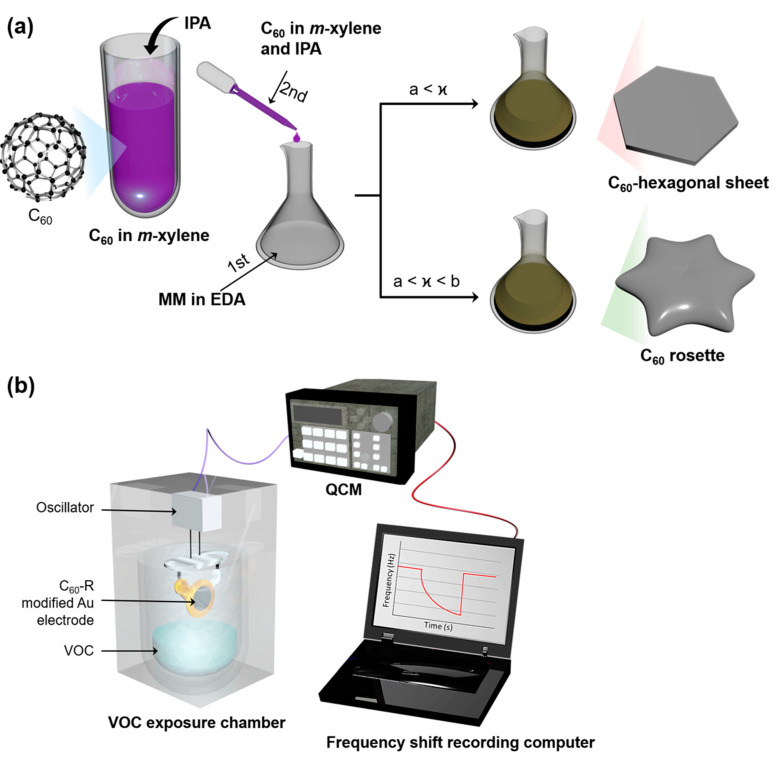
Schematics of the methods for (**a**) synthesis of 2D fullerene rosettes (C_60_−R) and (**b**) sensing of volatile organic compounds (VOCs); IPA = isopropyl alcohol, MM = melamine, EDA = ethylenediamine, and QCM = quartz crystal microbalance. The “ϰ” in panel (**a**) represents the amount of MM where only a certain ratio of MM (a < ϰ < b) resulted in the C_60_−R, and below the optimized amount of MM (a < ϰ) yielded the self-assembled hexagonal sheet.

In this work, we applied in the situ reactive method to the self-assembly process of C_60_ molecules with melamine/ethylenediamine components in solution (Figure 1a). As a novel fullerene assembly, fullerene rosettes, micron-sized two-dimensional, amorphous shape-regular objects, were successfully prepared. Structural tuning of the fullerene rosettes is here reported together with fundamental characterizations of the fullerene rosettes. In addition, prototype sensor systems (Figure 1b) were fabricated upon immobilization of the fullerene rosettes onto surfaces of a quartz crystal microbalance (QCM), and selective sensing of formic acid (HCOOH) was demonstrated as preliminary results for social-demanded toxic material sensing.

Formic acid is a small volatile organic compound (VOC) with irritant, corrosive, and caustic properties that are widely used as a preservative or antibacterial agent, raw materials for other compounds, leather-processing industries, and a hydrogen carrier for hydrogen production. Contact with formic acid vapor may cause external chemical burns, while inhalation can cause severe chemical pneumonitis, nerve injury, and dermatosis [42,43,44]. Thus, early detection of formic acid in the surrounding air is very important and the fabrication of an inexpensive, real-time sensor for monitoring formic acid would be useful not only for air-quality monitoring for medical diagnostics to secure our health, but also to limit formicary corrosion of industrial pipelines and equipment.

## 2. Results

### 2.1. Characterizations of the Prepared C_60_ Self-Assembled Structures

**Figure 2:**
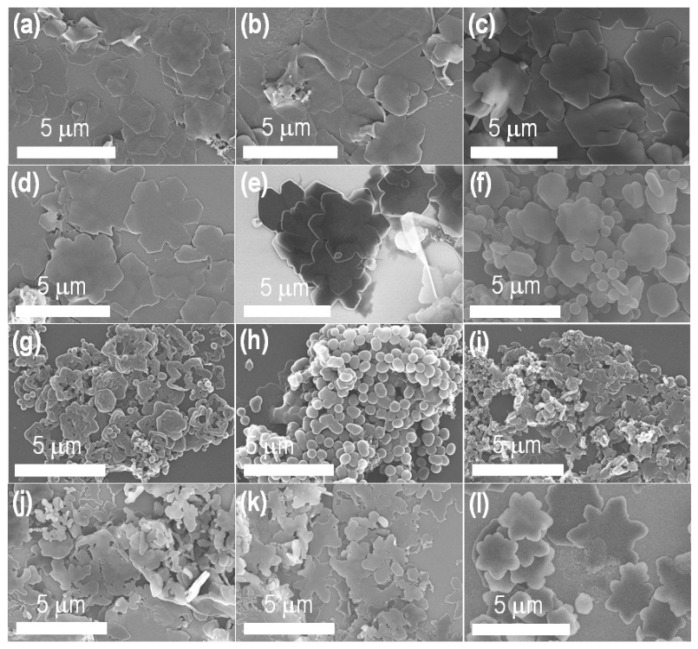
SEM images (**a**–**l**) of the materials obtained from the compositions listed in Table 1; entry 1–12, respectively.

To obtain fascinating C_60_ self-assembled structures with special chemical functionality, the liquid–liquid interface precipitation of C_60_ was carried out in the presence of ethylenediamine, melamine, and isopropyl alcohol using an *m*-xylene solution of pC_60_ under the ambient conditions. Table 1 summarizes the reaction compositions and surface morphology of the material obtained from each composition. Figure 2 shows the scanning electron microscopy (SEM) images of the materials that revealed the formation of 2D fullerene nanostructures with different morphologies/shapes under the studied conditions (Table 1). Interestingly, changing the precursor/solvent compositions can satisfactorily tune the shapes of the 2D nanostructures. In particular, hexagonal 2D fullerene nanosheet (Figure 2a) was obtained using the composition of pristine C_60_:isopropyl alcohol:melamine-ethylenediamine = 8.0:2.0:0.5 (*v*/*v*/*v*). However, by increasing the amount of melamine-ethylenediamine (entry 1–5), flower-like 2D nanostructures (Figure 2b–e) could be obtained. The composition of pC_60_:isopropyl alcohol:melamine-ethylenediamine = 8.0:2.0:1.0 (*v*/*v*/*v*) or increased melamine-ethylenediamine yields a beautiful 2D nanoflower morphology (Figure 2e; entry 5), indicating the role of melamine-ethylenediamine on the formation of 2D fullerene nanoflower, which can also be referred to as 2D fullerene rosette (C_60_−R).

The reactant compositions were further changed systematically to find the optimum condition for the beautiful 2D C_60_−R(entry 6–12; Table 1); for example, increasing the concentration of C_60_ solution and keeping a constant solvent ratio (entry 6), increases the amount of melamine-ethylenediamine solution (entry 7), and change the volume of C_60_ (from 8.0 to 8.5 mL) and isopropyl alcohol (2.0 to 1.5) keeping the melamine-ethylenediamine as same as entry 1 to 4. Figure 2f–i shows heterogeneous C_60_ self-assembled structures. In contrast, a 2D C_60_−R also could be obtained from the increased pC_60_ solution with decreased isopropyl alcohol when the melamine-ethylenediamine volume ranged from 0.7 to 0.8 mL. Thus far, the composition of pC_60_:isopropyl alcohol:melamine-ethylenediamine = 8.5:1.5:0.8 (*v*/*v*/*v*) yields the 2D fullerene rosettes with the most uniform shapes and sizes with distributions at submicrometer level.

**Figure 3:**
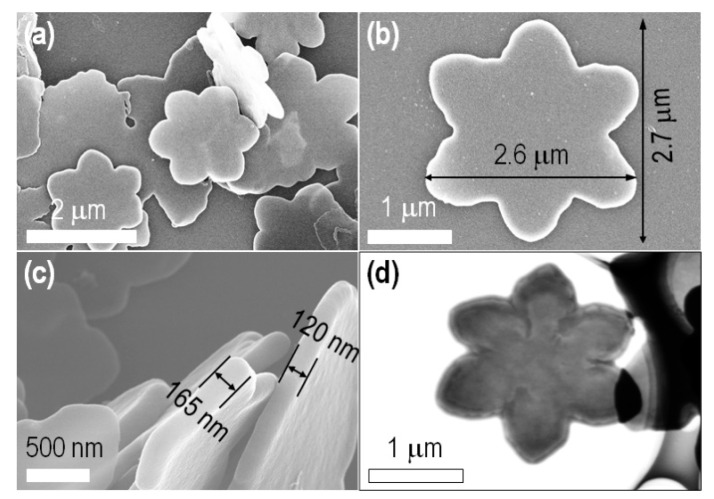
SEM images (**a**–**c**) and STEM image (**d**) of the 2D fullerene rosette. Morphology (**a**) and crystal dimension and surface texture (**b**,**c**).

**Figure 4:**
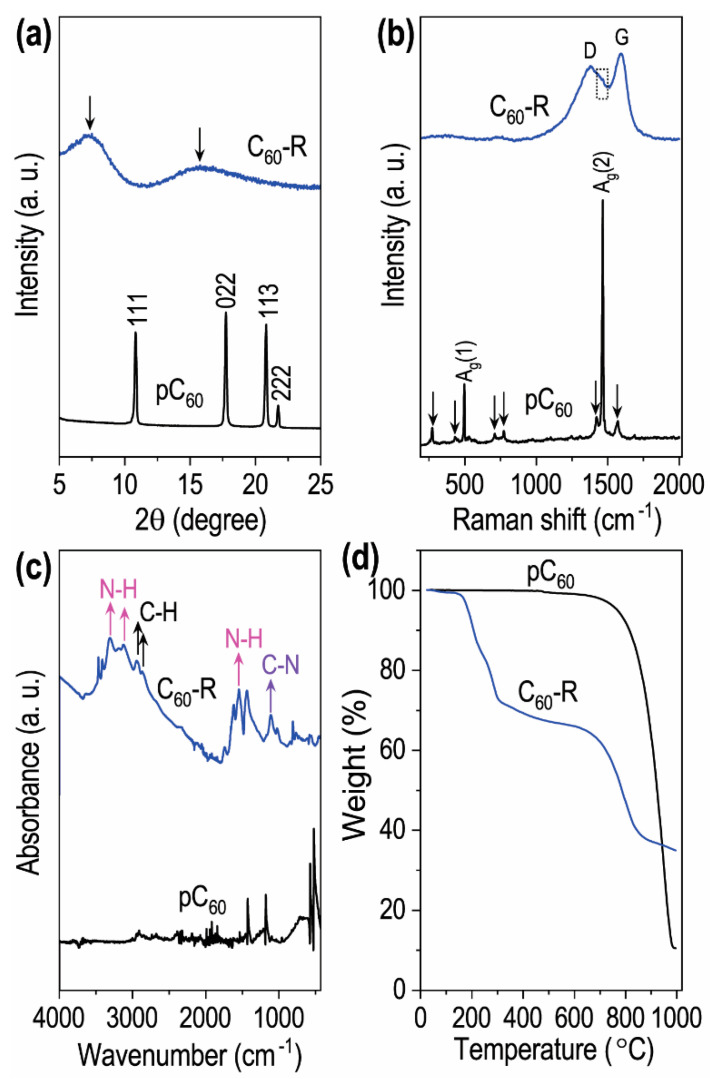
Characterizations of pC_60_ and the self-assembled C_60_−R: (**a**) powder X-ray diffraction patterns, (**b**) Raman scattering spectra, (**c**) FT–IR spectra, and (**d**) TGA curves.

The magnified SEM and scanning transmission electron microscopy (STEM) images, as shown in Figure 3, clearly reveal that the material obtained has a beautiful rosette shape. More precisely, the 2D C_60_−R consists of six petals, partially analogous to the hexagonal sheet (entry 1; Figure 2a), with a quite smooth surface having a thickness in the range 120–165 nm. As shown XRD patterns (Figure 4a), clear crystalline features within the 2D plane were not detected, suggesting mostly an amorphous nature in the 2D plane, which was also indicated in featureless texture within the magnified images.

The newly prepared C_60_−R was characterized using X-ray diffraction, Raman spectroscopy, FT–IR, TGA, and XPS techniques. The results were compared with pC_60_ to understand the change in pC_60_ under the self-assembled condition for the fullerene rosette. The XRD pattern of the C_60_−R was compared with that of the pC_60_ (Figure 4a). The diffraction pattern of C_60_−R shows two new broad diffraction bands at the diffraction angles (2θ) of approximately 7.3° and 15.9°, without any peaks for the crystalline pC_60_, indicating that the C_60_−R has an amorphous structure with the interlayer *d*-spacing value ca. 1.21 nm.

The Raman scattering spectrum (Figure 4b) of the C_60_−R has two intense *D* (defective) and *G* (graphitic) bands, respectively, at 1368 and 1585 cm^−1^, corresponding to the amorphous carbon. A small A_g_(2) band located at 1463 cm^−1^ for pC_60_ suggests that the free molecular rotation of fullerene molecules is restricted in the 2D C_60_−R due to strong interactions between melamine/ethylenediamine and fullerene molecules. The *I*_D_/*I*_G_ ratio of the 2D C_60_−R around ca. 0.85 is less than 1.0, suggesting an average abundance of defect distance [45]. Different from the pC_60_ fullerenes, the ATR–FTIR spectrum of the C_60_−R comprised broad N−H stretching vibrations at 3500–3150 cm^−1^; N–H deformation vibrations at 1634, 1546, and 1435 cm^−1^; and C–N stretching at 1250–1020 cm^−1^ (Figure 4c), which are similar to those of 1,2-ethylenediamine and melamine [46].

Thermogravimetric analysis (TGA), a tool widely used for evaluating thermal degradation behaviors of materials, was performed under the nitrogen atmosphere for C_60_−R and pC_60_, and the results are compared in Figure 4d. The TGA curves of the C_60_−R showed two-stage weight loss, a clear difference from that of the pC_60_ (single-stage degradation). First, weight loss was seen at approximately 150 to 300 °C, and the second was observed at around 750 °C.

**Figure 5:**
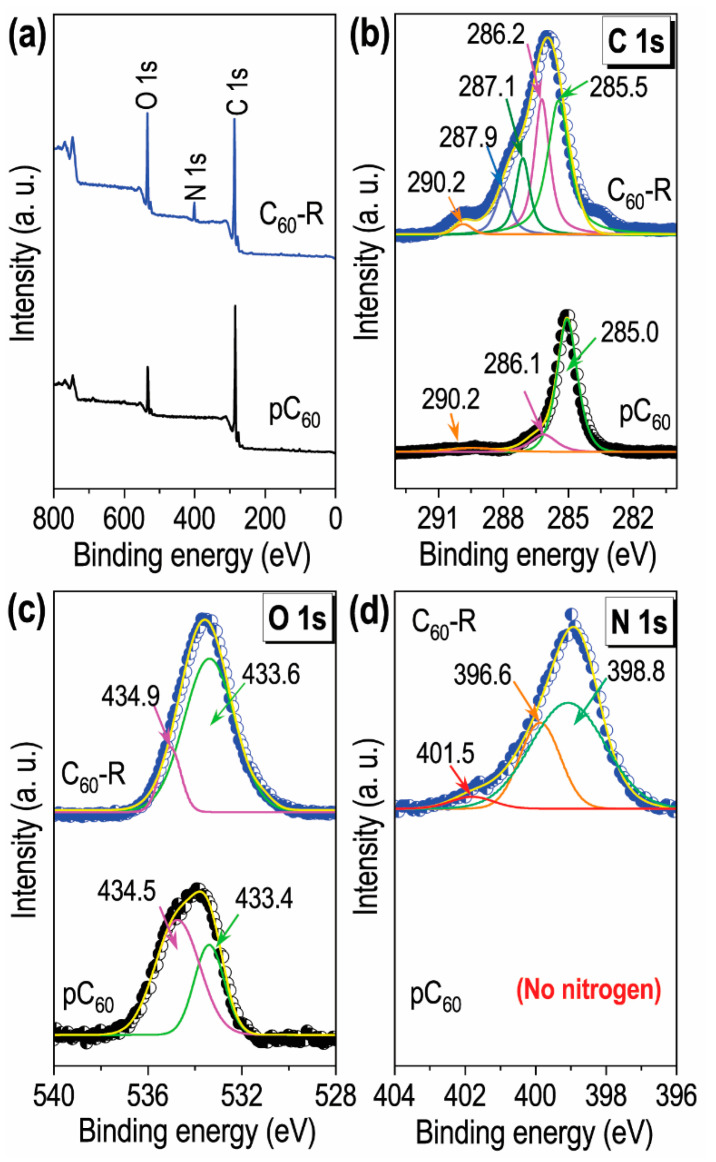
XPS studies of the C_60_−R and pC_60_: (**a**) XPS survey spectra; (**b**) XPS C 1s spectra with the deconvoluted peaks; (**c**) O 1s spectra with the deconvoluted peaks; and (**d**) N 1s spectrum with the deconvoluted peaks.

X-ray photoelectron spectroscopy (XPS) was carried out for a deeper understanding of the surface properties of the fullerene rosette. The XPS survey spectra (Figure 5a) of pC_60_ and C_60_−R indicate both are carbon with partial surface oxidation in which the latter one has an additional peak corresponding to nitrogen species. The carbon, oxygen, and nitrogen content in C_60_−R, analyzed from the XPS peaks, are 76.4, 18.6, and 5.0 atom%, respectively. The high-resolution C_1s_ spectrum (Figure 5b) of the pC_60_ show three distinct curves that peak at 285.0, 286.1, and 290.2 eV due to the C=C (sp^2^), C−C (sp^3^), and CO_3_^2−^ bonding states of carbon, while the C_60_−R showed five peaks at the binding energies 285.5, 286.6, 287.7, and 290.2, corresponding to C=C (sp^2^), C−C (sp^3^), C−N and CO_3_^2^ bonding states of carbon. The O 1s spectrum of pC_60_ can be deconvoluted into two peaks centered at 533.4 and 534.5 eV, corresponding to C−OH, and C−O−C bonding states, while the spectrum of the C_60_−R also could be deconvoluted into two peaks (Figure 5c) at the binding energies of 533.6, and 534.9 eV (with a slight shifting) for C−OH, and C−O−C bonding states of oxygen. The deconvoluted N 1s spectra of the C_60_−R shows existing nitrogen is in −NH− (398.8 eV), NH_2_ (399.6), and positively charged nitrogen (401.5) states [47] while pC_60_ does not have any nitrogen species.

### 2.2. Sensing of Organic Vapors by Quartz Crystal Microbalance

**Figure 6:**
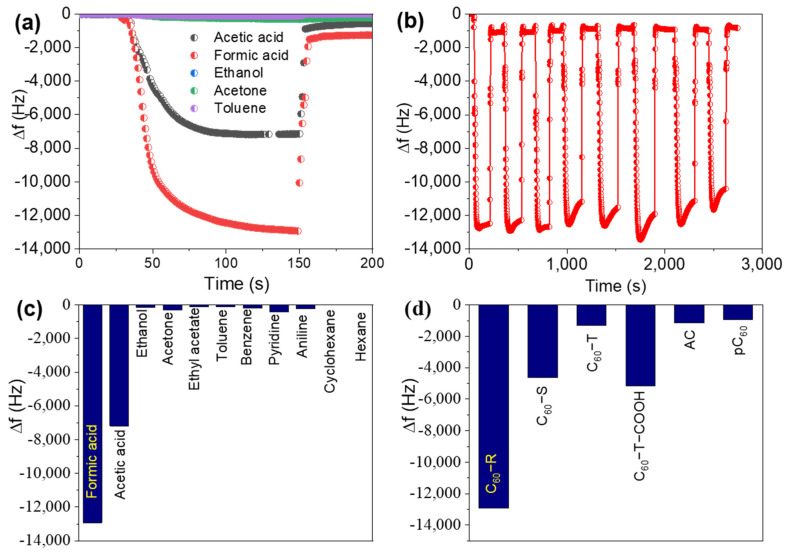
Vapor sensing performance of C_60_-R: (**a**) frequency shifts (∆f) upon exposure of QCM electrode to the vapors of different solvents (formic acid, acetic acid, acetone, toluene, and ethanol) as typical example; (**b**) repeatability test upon alternative exposure and removal of formic acid vapor; (**c**) relative sensing performance for various organic volatile vapors, and (**d**) comparison of the formic acid vapor sensing performances of different materials.

Newly developed unique 2D fullerene rosettes are enriched with various functionalities, especially nitrogen-containing amine or imine functionality, originating from the incorporated melamine and ethylenediamine, and thus have an attractive prospect to bind/sense guest molecules. As such, 2D fullerene rosettes were employed as sensor materials for a wide range of toxic volatile organic compounds (VOCs) such as acetic acid, formic acid, acetone, ethanol, ethyl acetate, benzene, toluene, pyridine, aniline, cyclohexane, and hexane by the quartz crystal microbalance (QCM) technique. Figure 6a presents the time−dependent frequency shifts (Δf) of the 2D fullerene rosette-modified QCM electrode upon exposure to a few typical VOC vapors: formic acid, acetic acid, acetone, ethanol, and toluene. A quick frequency shift was observed upon exposure to the VOCs, and the frequency returns nearly to the initial states upon removal of the solvent vapors from the chamber, suggesting reversible vapor adsorption/desorption. The repeatability test result obtained upon alternate exposure and removal of the formic acid vapor (Figure 6b) suggests excellent sensing performance of the fullerene rosette-modified QCM sensor with good repeatability. The sensing performance of a wide range of VOCs was measured over the pC_60_ and compared to that of the 2D fullerene rosettes (Figure 6c). The 2D fullerene rosettes always show higher sensitivity than the pC_60_ and the sensitivity generally follows: formic acid > acetic acid > pyridine > acetone > aniline > benzene > ethanol > ethyl acetate > toluene > cyclohexane > hexane. Moreover, formic acid, the representative volatile acid, sensing was performed over the QCM modified by some other fullerene self-assembled structures such as hexagonal nanosheet (C_60_−S; entry 1 of Table 1), fullerene nanotube (C_60_−T), acid-treated fullerene nanotube (C_60_−T−COOH), and commercial activated carbon (AC), for comparison. Interestingly, as shown in Figure 6d, the 2D fullerene rosette-modified QCM electrode showed remarkable performance compared to the other materials tested. Therefore, the 2D fullerene rosettes can be regarded as one of the best sensitive materials among the studied and reported materials (Table 2) for formic acid sensing using the QCM technique [40,41,48,49,50,51,52,53,54].

## 3. Discussion

The procedure combined with self-assembling and interaction with melamine/ethylenediamine resulted in separate two-dimensional objects with rosette-like shapes with thickness of about 120 nm. ATR–FTIR spectra, XPS, and TGA results suggest preservation of melamine/ethylenediamine components in C_60_−R assemblies even after washing with several solvents. The latter fact implies that the melamine/ethylenediamine components strongly interact and/or are covalently linked with fullerenes, as reported in previous examples [55,56]. Crystallinity indicated from XRD patterns implies significant differences in assembling mechanisms between pristine C_60_ crystals and C_60_−R. In contrast to various fullerene assemblies obtained from the liquid–liquid interfacial precipitation and related methods, the C_60_−R objects are noncrystalline amorphous. The broad peak for layer spacing in the XRD patterns of the C_60_−R objects corresponds roughly to the interdigitated fullerene bilayer or monolayer of modified fullerene molecules. This result indicates that the C_60_−R objects are made from an accumulation of bilayer/monolayer assemblies of hybridized fullerenes where fullerene rotations are limited upon intermolecular interactions but entire assemblies in low crystallinity. These two-dimensional layer features of fundamental structures are often recognized in assemblies of modified fullerene with amphiphilic natures. Interaction and possible hybridization with melamine/ethylenediamine components during the fullerene assembling process would result in two-dimensional amorphous assemblies. Instead of forming continuous two-dimensional films, separate shaped structures such as hexagons and rosettes, interestingly, were obtained in the current cases. This specific phenomenon would be related to the stability of edge formation. Favorable interactions of hybridized functional groups, mainly the amino group with surrounding solvents, may work to form separate size-limited two-dimensional objects instead of continuous two-dimensional growth. A balance between line tension at the edges and kinetic factors may determine the shapes of two-dimensional C_60_−R under various conditions.

Two-dimensional nature of the C_60_−R obtained would be advantageous for immobilization onto electrode-like device surfaces. Sensor application upon coating the electrode surface of QCM by two-dimensional C_60_−R would be an effective way for sensing VOC with portable use, such as simple exposures of the modified QCM devices to target vapors. Because fullerene molecules and their derivatives have rich π-electrons in *sp*^2^ carbons, the fullerene assemblies and related carbon materials exhibit higher sensitivity to vapors of aromatic guests such as benzene and toluene [57,58]. Several examples of fullerene-based QCM sensors with nonaromatic sensitivity are summarized in Table 2. Judging from apparent response values (frequency changes upon guest exposure, the QCM sensor with C_60_−R is categorized as one of the large-response sensors. However, these values are not collected under the unified conditions, parts of which are unknown in the literature. Therefore, the ratio of responses (ratio of response frequencies) is further compared. From viewpoints of selectivity to formic acids against basic guests or aromatic guests, the rosette-based QCM sensor shows superior performance. The QCM sensor with the C_60_−R objects has high capability for acid vapor sensing, probably because of the rich amino groups in their assemblies. Even for delicate size discrimination between formic acid and acetic acid, the rosette-based QCM sensor can be categorized as a better one. The highest selectivity between formic acid and acetic acid is observed for the previously reported fullerepnene-based sensor [41]. The latter one has nanometer-sized pores and thickness upon calcination at high temperatures that may work for better sensitivity. It must be noted that the current rosette-based sensor is capable of sensitive and selective detection for formic acids even though high-temperature processing can be avoided.

## 4. Materials and Methods

### 4.1. Materials

Pristine fullerene C_60_ (pC_60_: purity 99.9%) powder was bought from BBS Chemicals, Chimes Drive, Houston, TX, USA. Isopropyl alcohol (purity 99.7%), ethylenediamine (EDA), melamine, and *m*-xylene (purity 99.8%) were from the products of Wako Chemical Corporation, Tokyo, Japan.

### 4.2. Synthesis of C_60_ Rosettes

Solutions of fullerene C_60_ with desired concentrations (0.5 or 1.0 mg/mL) were prepared by dissolving the required amount of pC_60_ powder via sonication in *m*-xylene. Undissolved or excessive fullerene was removed by filtration when necessary. Separately, melamine in ethylenediamine solution (10.0 mg/mL) was also prepared via dissolving melamine into ethylenediamine by hand-shaking. The fullerene C_60_ self-assembled rosette crystals were synthesized by following the commonly used liquid–liquid interphase precipitation method. A certain amount of isopropyl alcohol was added into a glass vial (10.0 mL) containing the freshly prepared C_60_ solution in *m*-xylene and well-mixed by simple hand-shaking for about 5 s. The resulting mixture was then added quickly into melamine solution and immediately hand shaken for about 3 s. The slurry was incubated at 25 °C for 6 h without any external mechanical disturbances. The exact compositions of each solution/solvent for the synthesis of C_60_ self-assembled crystals are summarized in Table 1. The precipitates were separated from the mixture by centrifugation, followed by washing with isopropyl alcohol (5 mL) and deionized water (5.0 mL) three times to remove the organic solvents and melamine, and finally dried in an oven at 70 °C under vacuum for 6 h.

### 4.3. Characterizations

The materials obtained were characterized by using various techniques, including scanning electron microscopy (SEM, operating at 10 kV, Hitachi S-4800, Tokyo, Japan), scanning transmission electron microscopy (STEM, operating at 30 kV, Hitachi S-4800, Tokyo, Japan), powder X-ray diffraction (operated at 40 kV, Cu-Kα radiation (=0.1541 nm) RINT2000 diffractometer, Rigaku, Tokyo, Japan), Fourier transform infrared (FT–IR) spectroscopy (ATR–FTIR, Nexus 670, Tokyo, Japan), Raman scattering (NRS-3100 Raman spectrometer, JASCO, Tokyo, Japan), and X-ray photoelectron spectroscopy (XPS; Thermo Electron Co. Karlsruhe, Germany, a monochromatic Al-Kα radiation of photon energy 15 keV). The electron microscopy samples were prepared on carbon-coated copper grids by dropping C_60_ self-assembled crystals (the selected ones) suspension in isopropyl alcohol (3 μL) and drying under vacuum at 70 °C.

### 4.4. Sensing of Vapor by Quartz Crystal Microbalance (QCM)

The vapor sensing property of the studied materials was carried out using the QCM technique. We monitored the frequency shift in the Au-resonator decorated with the materials as QCM electrodes that were exposed to different organic vapors by a resonance frequency of 9 MHz (AT-cut). Notably, the stability of the QCM electrode was ±2 Hz in the air for 10 min. The method of making a QCM sensor electrode follows: solid materials excluding pC_60_ (1 mg) were dispersed in isopropyl alcohol (IPA, 1 mL) via sonication for 30 s, the suspension (2 μL) was then drop cast onto the Au resonator electrode. For the pC_60_, to obtain an accurate frequency shift on QCM, C_60_ saturated *m*-xylene solution (5 μL; relatively large volume) was drop cast onto the electrode. The as-prepared electrodes were dried at 70 °C under a vacuum for 5 h. The final QCM electrode was then plugged into the instrument and then exposed to the studied volatile organic solvents (10 mL in an open container) at room temperature. The chamber was immediately sealed to minimize escape of the vapors and to create a saturated vapor atmosphere during the frequency monitoring. Once the frequency reached equilibrium, the chamber was opened for vapor desorption.

## 5. Conclusions

In this work, we applied an in situ reactive method to the self-assembly process of C_60_ molecules with melamine/ethylenediamine components in solution. As a novel-type of fullerene assemblies, micron-sized two-dimensional, amorphous shape-regular objects, fullerene rosettes (C_60_−Rs), were successfully prepared. Structural tuning of the fullerene rosettes is reported together with fundamental characterizations of the fullerene rosettes. 

ATR–FTIR spectra, XPS, and TGA results suggest that the melamine/ethylenediamine components strongly interact and/or are covalently linked with fullerenes. In contrast to various fullerene assemblies obtained from liquid–liquid interfacial precipitation and other related methods, the fullerene rosettes are noncrystalline amorphous. The broad peak for layer spacing in the XRD patterns of the C_60_−R objects corresponds roughly to interdigitated fullerene bilayer or monolayer of modified fullerene molecules. This result indicates that the C_60_−R objects are made from an accumulation of bilayer/monolayer assemblies of hybridized fullerenes where fullerene rotations are limited upon intermolecular interactions but entire assemblies in low crystallinity. Prototype sensor systems were fabricated upon immobilization of the fullerene rosettes onto surfaces of a quartz crystal microbalance (QCM), and selective sensing of formic acid was demonstrated as preliminary results for social-demanded toxic material sensing. Judging from the apparent response values (frequency changes upon guest exposure), the QCM sensor with C_60_−R is categorized as one of the large-response sensors among the reported examples. From the viewpoints of selectivity to formic acids against basic guests or aromatic guests, the rosette-based QCM sensor also showed superior performance. The QCM sensor with the C_60_−R objects has high capabilities for acid vapor sensing, probably because of the rich amino groups in their assemblies.

It must be noted that the current rosette-based sensor is capable of sensitive and selective detection for formic acids even though high-temperature processing can be avoided. As described in the previous literature [59], sensors for formic acids and related compounds are required for tracing invasive formicine ant species [60], air quality monitoring [61,62], and health condition diagnostics [63,64,65]. Because the methodologies presented in this research are relatively simple, more advanced sensing systems can be constructed for various chemical targets, including these important VOCs. Although here we selected a QCM device as a conventional sensor system, more advanced sensor devices [66] can be applied. Simple and convenient QCM sensors demonstrated in this work can mainly give direction for the usages of the fullerene rosettes, but further applications of the fullerene rosettes to more advanced sensor devices with gas-flow control apparatus [66] will provide much better capabilities for toxic VOCs sensing with good limit of detections. Emerging concepts for material design such as material nanoarchitectonics [67,68] and material informatics [69] can be used for fabrications for sensing materials along with traditional techniques such as the Langmuir–Blodgett method [70] and later-by-later assembly [71] for material–sensor interfacing. Combinations of the materials design and system integration would create advanced sensing systems only using simple molecules such as fullerenes. In addition, further applications of the fullerene rosettes to electrophysics and photophysics can be considered on the basis of high capabilities of fullerenes in these fields [72,73].

## Figures and Tables

**Table 1:** Compositions of the liquid interfacial self-assembly of fullerene C_60_ crystals in the presence of isopropyl alcohol (IPA) and melamine (MM) using the *m*-xylene and ethylenediamine solution of C_60_ and MM, respectively. The illustrated shapes of the materials along with remarks are also provided.

Entry	C_60_in *m*-xylene (mL) ^1^	IPA (mL)	MM in EDA (mL)	Shapes	Remarks
1	8.0	2.0	0.5	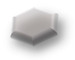	Edge tends to cleave
2	8.0	2.0	0.6	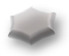	Edge cleaving started
3	8.0	2.0	0.7	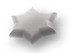	Edge cleaving increases
4	8.0	2.0	0.8	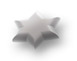	Edge cleaving increases more
5	8.0	2.0	1.0	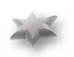	Beautiful flower shape formed
6	8.0 ^2^	2.0	1.0	−	No edge cleaving/heterogeneous shapes
7	8.0	1.0	2.0	−	No edge cleaving/heterogeneous shapes
8	8.0	1.0	2.0 ^3^	−	Heterogeneous shapes
9	8.5	1.5	0.5	−	Heterogeneous shapes
10	8.5	1.5	0.6	−	Heterogeneous shapes with sheet types
11	8.5	1.5	0.7	−	Heterogeneous flower shapes
12	8.5	1.5	0.8	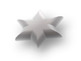	Most perfect and homogeneous beautiful flower shape formed

^1^ Concentration of C_60_ in *m*-xylene and melamine (MM) in EDA were 0.5 and 10.0 mg/L, respectively. ^2^ C_60_ concentration was 1.0 rather than 0.5 mg/mL. ^3^ MM in EDA concentration was 25.0 rather than 10.0 mg/mL.

**Table 2:** The formic acid sensing performances over the quartz crystal microbalance sensor modified with different materials. Abbreviation of terminology used in Table 1 is shown as a footnote ^1^.

Materials	ΔF (Hz)	ΔF (Hz) Ratio of FA/PRD (Acid/Base)	ΔF (Hz) Ratio of FA/AA (Acid/Acid)	ΔF (Hz) Ratio of FA/Hydrocarbon (Acid/Neutral)	Reference
PANI−QCM	20	−	−	−	[48]
DAP−QCM	824	−	−	−	[49]
MWCNT	196	−	1.3	−	[50]
MCN−ATN	1195	~4	1.16	10.5 (toluene)	[51]
Ph−g−C_3_N_4_	13,417	15.8	3.1	106.5 (benzene)	[52]
MOF−GC@COF	98	~2 (Et_3_N)	−	12.0 (*n*-hexane)	[53]
FNR−EDA	1758	~17	1.45	14.9 (toluene)	[40]
CHFC	1700	7.7	0.69	30.7 (toluene)	[54]
C_60_−fullerphene	21,204	−	18.8	375 (benzene)	[41]
C_60_−R	12,930	31.5	1.8	112 (toluene)	This study

^1^ PANI−QCM = polyaniline-coated quartz crystal microbalance sensor; DAP−QCM = 2,6-diacetylpyridine coated quartz crystal microbalance sensor; MWCNT: acidified multiwalled carbon nanotube film; MCN−ATN = mesoporous carbon nitride from 3-amino-1,2,4-triazine; Ph-g-C_3_N_4_ = phenyl-terminated carbon nitride quantum nanoflakes; MOF−GC@COF = metal–organic framework-derived graphitic carbon core and a well-arranged covalent organic framework shell; FNR = fullerene nanorods; EDA = ethylene diamine; CHFC = corn-husk-shaped fullerene C_60_ crystals; C_60_-fullerphene = nitrogen-doped 2D fullerphene; C_60_−R = fullerene rosette; FA = formic acid; PRD = pyridine; AA = acetic acid.

## Data Availability

Not applicable.

## References

[1] Loll B., Kern J., Saenger W., Zouni A., Biesiadka J. (2005). Towards complete cofactor arrangement in the 3.0 Å resolution structure of photosystem II. Nature.

[2] Wang J., Song L., Gong X., Xu J., Li M. (2020). Functions of jasmonic acid in plant regulation and response to abiotic stress. Int. J. Mol. Sci..

[3] Voorn R.A., Vogl C. (2020). Molecular assembly and structural plasticity of sensory ribbon synapses—A presynaptic perspective. Int. J. Mol. Sci..

[4] Whitesides G.M., Mathias J.I., Seto C.T. (1991). Self-assembly and nanochemistry: A chemical strategy for the synthesis of nanostructures. Science.

[5] Wasielewski M.R. (2009). Self-assembly strategies for integrating light harvesting and charge separation in artificial photosynthetic systems. Acc. Chem. Res..

[6] Percec V., Xiao Q. (2021). Helical self-organizations and emerging functions in architectures, biological and synthetic macromolecules. Bull. Chem. Soc. Jpn..

[7] Ariga K., Shrestha L.K. (2021). Zero-to-one (or more) nanoarchitectonics: How to produce functional materials from zero-dimensional single-element unit, fullerene. Mater. Adv..

[8] Chen G., Shrestha L.K., Ariga K. (2021). Zero-to-two nanoarchitectonics: Fabrication of two-dimensional materials from zero-dimensional fullerene. Molecules.

[9] Maji S., Shrestha L.K., Ariga K. (2021). Nanoarchitectonics for hierarchical fullerene nanomaterials. Nanomaterials.

[10] Babu S.S., Möhwald H., Nakanishi T. (2010). Recent progress in morphology control of supramolecular fullerene assemblies and its applications. Chem. Soc. Rev..

[11] Das S., Presselt M. (2019). Progress and development in structural and optoelectronic tunability of supramolecular nonbonded fullerene assemblies. J. Mater. Chem. C.

[12] Han F., Wang R., Feng Y., Wang S., Liu L., Li X., Han Y., Chen H. (2019). On demand synthesis of hollow fullerene nanostructures. Nat. Commun..

[13] Xu Y., Guo J., Wei T., Chen X., Yang Q., Yang S. (2013). Micron-sized hexagonal single-crystalline rods of metal nitride clusterfullerene: Preparation, characterization, and photoelectrochemical application. Nanoscale.

[14] Ji H.-X., Hu J.-S., Tang Q.-X., Song W.-G., Wang C.-R., Hu W.-P., Wan L.-J., Lee S.-T. (2007). Controllable preparation of submicrometer single-crystal C_60_ rods and tubes trough concentration depletion at the surfaces of seeds. J. Phys. Chem. C.

[15] Zheng S., Cuong N.T., Okada S., Xu T., Shen W., Lu X., Tsukagoshi K. (2018). Solvent-mediated shape engineering of fullerene (C_60_) polyhedral microcrystals. Chem. Mater..

[16] Miyazawa K. (2015). Synthesis of fullerene nanowhiskers using the liquid–liquid interfacial precipitation method and their mechanical, electrical and superconducting properties. Sci. Technol. Adv. Mater..

[17] Minato J., Miyazawa K. (2005). Solvated structure of C_60_ nanowhiskers. Carbon.

[18] Sathish M., Miyazawa K., Sasaki T. (2007). Nanoporous fullerene nanowhiskers. Chem. Mater..

[19] Jin Y., Curry R.J., Sloan J., Hatton R.A., Chong L.C., Blanchard N., Stolojan V., Kroto H.W., Silva S.R.P. (2006). Structural and optoelectronic properties of C_60_ rods obtained via a rapid synthesis route. J. Mater. Chem..

[20] Somani P.R., Somani S.P., Umeno M. (2007). Toward organic thick film solar cells: Three dimensional bulk heterojunction organic thick film solar cell using fullerene single crystal nanorods. Appl. Phys. Lett..

[21] Minato J., Miyazawa K., Suga T. (2005). Morphology of C_60_ nanotubes fabricated by the liquid–liquid interfacial precipitation method. Sci. Technol. Adv. Mater..

[22] Miyazawa K., Minato J., Yoshii T., Fujino M., Suga T. (2005). Structural characterization of the fullerene nanotubes prepared by the liquid–liquid interfacial precipitation method. J. Mater. Res..

[23] Sathish M., Miyazawa K. (2007). Size-tunable hexagonal fullerene (C_60_) nanosheets at the liquid−liquid interface. J. Am. Chem. Soc..

[24] Wakahara T., D’Angelo P., Miyazawa K., Nemoto Y., Ito O., Tanigaki N., Bradley D.D.C., Anthopoulos T.D. (2012). Fullerene/cobalt porphyrin hybrid nanosheets with ambipolar charge transporting characteristics. J. Am. Chem. Soc..

[25] Osonoe K., Kano R., Miyazawa K., Tachibana M. (2014). Synthesis of C_70_ two-dimensional nanosheets by liquid–liquid interfacial precipitation method. J. Cryst. Growth.

[26] Park C., Yoon E., Kawano M., Joo T., Choi H.C. (2010). Self-Crystallization of C_70_ cubes and remarkable enhancement of photoluminescence. Angew. Chem. Int. Ed..

[27] Hill J.P., Shrestha R.G., Song J., Ji Q., Ariga K., Shrestha L.K. (2021). Monitoring the release of silver from a supramolecular fullerene C_60_-AgNO_3_ Nanomaterial. Bull. Chem. Soc. Jpn..

[28] Partheeban T., Sathish M. (2016). Selective growth of fullerene octahedra and flower-like particles by a liquid–liquid interfacial precipitation method for super-hydrophobic applications. RSC Adv..

[29] Chen N., Hu Y., Xu T., Lu X. (2020). Three-dimensional “star of David”-shaped fullerene (C_60_) microstructures: Controlled synthesis, photoluminescence, and photoelectrochemical properties. ACS Appl. Electron. Mater..

[30] Bairi P., Minami K., Hill J.P., Ariga K., Shrestha L.K. (2017). Intentional closing/opening of “hole-in-cube” fullerene crystals with microscopic recognition properties. ACS Nano.

[31] Bairi P., Minami K., Nakanishi W., Hill J.P., Ariga K., Shrestha L.K. (2016). Hierarchically structured fullerene C_70_ cube for sensing volatile aromatic solvent vapors. ACS Nano.

[32] Maji S., Shrestha R.G., Lee J., Han S.A., Hill J.P., Kim J.H., Ariga K., Shrestha L.K. (2021). Macaroni fullerene crystals-derived mesoporous carbon tubes as a high rate performance supercapacitor electrode material. Bull. Chem. Soc. Jpn..

[33] Tang Q., Maji S., Jiang B., Sun J., Zhao W., Hill J.P., Ariga K., Fuchs H., Ji Q., Shrestha L.K. (2019). Manipulating the structural transformation of fullerene microtubes to fullerene microhorns having microscopic recognition properties. ACS Nano.

[34] Nakanishi T., Michinobu T., Yoshida K., Shirahata N., Ariga K., Möhwald H., Kurth D.G. (2008). Nanocarbon superhydrophobic surfaces created from fullerene-based hierarchical supramolecular assemblies. Adv. Mater..

[35] Nakanishi T., Shen Y., Wang J., Li H., Fernandes P., Yoshida K., Yagai S., Takeuchi M., Ariga K., Kurth D.G. (2010). Superstructures and superhydrophobic property in hierarchical organized architectures of fullerenes bearing long alkyl tails. J. Mater. Chem..

[36] Zhou S., Burger C., Chu B., Sawamura N., Nagahama N., Toganoh M., Hackler U.E., Isobe H., Nakamura E. (2001). Spherical bilayer vesicles of fullerene-based surfactants in water: A laser light scattering study. Science.

[37] Neal E.A., Nakanishi T. (2021). Alkyl-fullerene materials of tunable morphology and function. Bull. Chem. Soc. Jpn..

[38] Bairi P., Minami K., Hill J.P., Nakanishi W., Shrestha L.K., Liu C., Harano K., Nakamura E., Ariga K. (2016). Supramolecular differentiation for construction of anisotropic fullerene nanostructures by time-programmed control of interfacial growth. ACS Nano.

[39] Michinobu T., Nakanishi T., Hill J.P., Funahashi M., Ariga K. (2006). Room temperature liquid fullerenes:  an uncommon morphology of C_60_ derivatives. J. Am. Chem. Soc..

[40] Hsieh C.-H., Hsu S.-H., Maji S., Chahal M.K., Song J., Hill J.P., Ariga K., Shrestha L.K. (2020). Post-assembly dimension-dependent face-selective etching of fullerene crystals. Mater. Horiz..

[41] Song J., Murata T., Tsai K.-C., Jia X., Sciortino F., Ma R., Yamauchi Y., Hill J.P., Shrestha L.K., Ariga K. (2022). Fullerphene nanosheets: A bottom-Up 2D material for single-carbon-atom-level molecular discrimination. Adv. Mater. Interfaces.

[42] Davis D., Kundu S., Prabhudesai V.S., Sajeev Y., Krishnakumar E. (2018). Formation of CO_2_ from formic acid through catalytic electron channel. J. Chem. Phys..

[43] Mori K., Dojo M., Yamashita H. (2013). Pd and Pd−Ag nanoparticles within a macroreticular basic resin: An efficient catalyst for hydrogen production from formic acid decomposition. ACS Catal..

[44] Lu N., Gao X., Yang C., Xiao F., Wang J., Su X. (2016). Enhanced formic acid gas-sensing property of WO_3_ nanorod bundles via hydrothermal method. Sens. Actuators B.

[45] Toh C.T., Zhang H., Lin J., Mayorov A.S., Wang Y.P., Orofeo C.M., Ferry D.B., Andersen H., Kakenov N., Guo Z. (2020). Synthesis and properties of free-standing monolayer amorphous carbon. Nature.

[46] Zhu H., Xu S.A. (2018). Preparation and fire behavior of rigid polyurethane foams synthesized from modified urea-melamine-formaldehyde resins. RSC Adv..

[47] Lee M.S., Park M., Kim H.Y., Park S.J. (2016). Effects of microporosity and surface chemistry on separation performances of N-containing pitch-based activated carbons for CO_2_/N_2_ binary mixture. Sci. Rep..

[48] Yan Y., Lu D., Zhou H., Hou H., Zhang T., Wu L., Cai L. (2012). Polyaniline-modified quartz crystal microbalance sensor for detection of formic acid gas. Water Air Soil Pollut..

[49] Leyva J.A.M., de Cisneros J.L.H.H., de Barreda D.G.G., Becerra A.J.F. (1993). Determination of formic acid vapour using piezoelectric crystals with 4-ethyl-3-thiosemicarbazide and 2,6-diacetylpyridine coatings. Analyst.

[50] Cai J., Yan Y., Wang W., Ma Y., Cai L., Wu L., Zhou H. (2021). Detection of formic acid and acetic acid gases by a QCM sensor coated with an acidified multi-walled carbon nanotube membrane. Environ. Technol..

[51] Mane G.P., Dhawale D.S., Anand C., Ariga K., Ji Q., Wahab M.A., Mori T., Vinu A. (2013). Selective sensing performance of mesoporous carbon nitride with a highly ordered porous structure prepared from 3-amino-1,2,4-triazine. J. Mater. Chem. A.

[52] Torad N.L., El-Hosainy H., Esmat M., El-Kelany K.E., Tahawy R., Na J., Ide Y., Fukata N., Chaikittisilp W., Hill J.P. (2021). Phenyl-modified carbon nitride quantum nanoflakes for ultra-highly selective sensing of formic acid: A combined experimental by QCM and density functional theory study. ACS Appl. Mater. Interfaces.

[53] Zhang S., Yang Q., Xu X., Liu X., Li Q., Guo J., Torad N.L., Alshehri S.M., Ahamad T., Hossain M.S.A. (2020). Assembling well-arranged covalent organic frameworks on MOF-derived graphitic carbon for remarkable formaldehyde sensing. Nanoscale.

[54] Wei Z., Song J., Ma R., Ariga K., Shrestha L.K. (2022). Self-assembled corn-husk-shaped fullerene crystals as excellent acid vapor sensors. Chemosens.

[55] Rašović I. (2017). Water-soluble fullerenes for medical applications. Mater. Sci. Technol..

[56] Sun Y., Cao C., Liu C., Liu J., Zhu Y., Wang X., Song W. (2017). Nitrogen-doped hollow carbon spheres derived from amination reaction of fullerene with alkyl diamines as a carbon catalyst for hydrogenation of aromatic nitro compounds. Carbon.

[57] Shrestha L.K., Shrestha R.G., Yamauchi Y., Hill J.P., Nishimura T., Miyazawa K., Kawai T., Okada S., Wakabayashi K., Ariga K. (2015). Nanoporous carbon tubes from fullerene crystals as the π-electron carbon source. Angew. Chem. Int. Ed..

[58] Furuuchi N., Shrestha R.G., Yamashita Y., Hirao T., Ariga K., Shrestha L.K. (2019). Self-assembled fullerene crystals as excellent aromatic Vapor Sensors. Sensors.

[59] Lin S., Swager T.M. (2018). Carbon nanotube formic acid sensors using a nickel bis(*ortho*-diiminosemiquinonate) selector. ACS Sens..

[60] Wang Z., Moshman L., Kraus E., Wilson B., Acharya N., Diaz R. (2016). A review of the Tawny Crazy Ant, Nylanderia Fulva, an emergent ant invader in the southern united states: Is biological control a feasible management option?. Insects.

[61] Stavrakou T., Müller J.-F., Peeters J., Razavi A., Clarisse L., Clerbaux C., Coheur P.-F., Hurtmans D., De Mazière M., Vigouroux C. (2011). Satellite evidence for a large source of formic acid from boreal and tropical forests. Nat. Geosci..

[62] Ishihara S., Labuta J., Nakanishi T., Tanaka T., Kataura H. (2017). Amperometric detection of sub-ppm formaldehyde using single-walled carbon nanotubes and hydroxylamines: A referenced chemiresistive system. ACS Sens..

[63] McMartin K.E., Ambre J.J., Tephly T.R. (1980). Methanol poisoning in human subjects. Role for formic acid accumulation in the metabolic acidosis. Am. J. Med..

[64] Greenwald R., Fitzpatrick A.M., Gaston B., Marozkina N.V., Erzurum S., Teague W.G. (2010). Breath formate is a marker of airway *S*-nitrosothiol depletion in severe asthma. PLoS ONE.

[65] Greenwald R., Johnson B.A., Hoskins A., Dworski R. (2013). Exhaled breath condensate formate after inhaled allergen provocation in atopic asthmatics in vivo. J. Asthma.

[66] Nishikawa M., Murata T., Ishihara S., Shiba K., Shrestha L.K., Yoshikawa G., Minami K., Ariga K. (2021). Discrimination of methanol from ethanol in gasoline using a membrane-type surface stress sensor coated with copper(I) complex. Bull. Chem. Soc. Jpn..

[67] Ariga K. (2021). Nanoarchitectonics: What’s coming next after nanotechnology?. Nanoscale Horiz..

[68] Ariga K., Fakhrullin R. (2022). Materials nanoarchitectonics from atom to living cell: A method for everything. Bull. Chem. Soc. Jpn..

[69] Chaikittisilp W., Yamauchi Y., Ariga K. (2022). Material evolution with nanotechnology, nanoarchitectonics, and materials informatics: What will be the next paradigm shift in nanoporous materials?. Adv. Mater..

[70] Oliveira O.N., Caseli L., Ariga K. (2022). The past and the future of Langmuir and Langmuir–Blodgett films. Chem. Rev..

[71] Ariga K., Decher G., Lvov Y. (2022). There is still plenty of room for layer-by-layer assembly for constructing nanoarchitectonics-based materials and devices. Phys. Chem. Chem. Phys..

[72] Baskar A.V., Ruban A.M., Davidraj J.M., Singh G., Al-Muhtaseb A.H., Lee J.M., Yi J., Vinu A. (2021). Single-step synthesis of 2D mesoporous C_60_/carbon hybrids for supercapacitor and Li-ion battery applications. Bull. Chem. Soc. Jpn..

[73] Santiago A.R.P., Fernandez-Delgado O., Gomez A., Ahsan M.A., Echegoyen L. (2021). Fullerenes as key components for low-dimensional (photo)electrocatalytic nanohybrid materials. Angew. Chem. Int. Ed..

